# Cyclin E1-CDK 2, a potential anticancer target

**DOI:** 10.18632/aging.100946

**Published:** 2016-04-14

**Authors:** Dongdong Fang, Shuang Huang, Shi-Bing Su

**Affiliations:** Research Center for Traditional Chinese Medicine Complexity System, Shanghai University of Traditional Chinese Medicine, Shanghai 201203, China

**Keywords:** cyclin-dependent kinase 2, ovary tumor, anticancer, target

CCNE1 is one of the most frequent gene amplifications in high grade serous ovarian cancers (HGSOCs), which occurs in at least 20% of HGSOC and in 30% of established ovarian cancer cell lines. Studies with both primary and metastatic ovary tumor specimens further suggest that the abundance of CCNE1 protein expression correlates with tumor progression and predicts a poor prognosis in ovarian cancer patients[1]. It is noted that, CCNE1 is critical for the growth of ovarian cancer cell lines with elevated CCNE1 expression but not cells without CCNE1 overexpression [1]. Furthermore, CCNE1 gene amplification-associated CCNE1 overexpression has been linked to the development of chemo-resistance in ovarian cancer[1]. Thus, accumulated findings implicate that CCNE1 may be a promising therapeutic target for ovary tumors with elevated CCNE1 expression. However, developing small molecules to target CCNE1 directly is unlikely, because CCNE1 acts as a regulatory subunit of cyclin-dependent kinase (Cdk) complex rather than as an enzyme or receptor.

It is well known that CCNE1 mainly coordinates with Cdk2 to facilitate G1/S progression of cell cycle. In ovary tumors, elevated CCNE1 level is often correlated with higher Cdk2 expression and most of CCNE1-associated tumor promoting effects require the participation of Cdk2. Thus, targeting Cdk2 may be an attractive alternative given the current availability of small molecule Cdk2 inhibitors. SNS-032 (BMS-387032) is a selective inhibitor of CDK2, and has been evaluated in Phase I study for patients with either chronic lymphocytic leukemia or multiple myeloma, as well as clinical safety assessment for the treatment of select advanced solid tumors. We showed that ovarian cancer cells with elevated CCNE1 expression are at least 40 times more sensitive to SNS-032 than those without CCNE1 overexpression. Moreover, we demonstrated that SNS-032 effectively suppresses the tumorigenicity of ovarian cancer cells by prolonging the survival of animals bearing tumors derived from ovarian cancer cells with elevated CCNE1 expression and inhibiting peritoneal metastatic colonization. These results suggest that ovary tumors with elevated CCNE1 expression may be staged for Cdk2-targeted therapy.

How about the potential usage of CDK2 inhibitor in other types of cancer? The significance of cyclin E amplification and overexpression in breast cancer has already been highlighted in serial studies. An interesting finding shows that in some breast, as well as in ovarian tumors, full-length (FL) cyclin E proteolytically be cleaved by the protease elastase, leading to low molecular weight (LMW) forms [2]. The group of K. Keyomarsi and K. Hunt [2] have discovered that HER2-positive breast cancer patients can be divided in two groups with different outcomes, which are FL-cyclin E type with the high survival rate and LMW-cyclin E type with low survival rate. The LMW-cyclin E thus may be used to differentiate and select patients for combined treatment with Trastuzumab for anti-HER2 and CDK2 inhibitors. Consistent with this finding, Maurizio Scaltriti et al. [3] further revealed that cyclin E amplification/overexpression is a mechanism of tras-tuzumab resistance in HER2+ breast cancer patients, and treatment with CDK2 inhibitors may be a valid strategy in patients with breast tumors with HER2 and cyclin E coamplification/overexpression. These findings indicated that CDK2 inhibitors may possess the potential to be combined with other strategies to overcome tumor drug resistance. In clinical settings SNS-032 was evaluated in patients with advanced chronic lymphocytic leukemia, multiple myeloma and advanced solid tumors. Another potent CDK inhibitor dinaciclib (SCH 727965) is under investigation in phase 1/2 clinical trial in patients with stage III-IV malignant melanoma.

Preclinical and clinical researches have pointed to the significance of Cyclin E-CDK2 signal as ideal targets for anti-neoplastic therapy both for used alone or combination application for increasing drug sensitivity. Although present focus is mainly on breast, ovarian cancer and melanoma, amplification and overexpression of Cyclin E was also observed in other cancer, including bladder [4], gastric [5] and colorectal cancer [6], and its correlation with prognosis was shown. Thus, further steps are needed to explore the potential of CDK2 inhibitors in a wider scope of anticancer usage, and amplification of Cyclin E may present as a target for precision cancer therapy.
